# New distribution records of *Aedes aegypti* (Linnaeus), *Aedes mediovittatus* (Coquillett), and *Toxorhynchites portoricensis* (Röder) (Diptera: Culicidae) in Puerto Rico and their relevance to integrated vector management

**DOI:** 10.1093/jme/tjag048

**Published:** 2026-03-27

**Authors:** Jun Soo Bae, Telmah Telmadarrehei, Sangwoo Seok, Lianmarie Soto Jiménez, Amaury Morales González, Luis F Quintanilla Vásquez, Valerie T Nguyen, Riley Young, Raymond Gellner, Lawrence E Reeves, Joanelis Medina, Grayson Brown, Yoosook Lee

**Affiliations:** Florida Medical Entomology Laboratory, Entomology and Nematology Department, Institute of Food and Agricultural Sciences, University of Florida, Vero Beach, FL, USA; Florida Medical Entomology Laboratory, Entomology and Nematology Department, Institute of Food and Agricultural Sciences, University of Florida, Vero Beach, FL, USA; Florida Medical Entomology Laboratory, Entomology and Nematology Department, Institute of Food and Agricultural Sciences, University of Florida, Vero Beach, FL, USA; Puerto Rico Vector Control Unit, San Juan, PR, USA; Puerto Rico Vector Control Unit, San Juan, PR, USA; Puerto Rico Vector Control Unit, San Juan, PR, USA; Florida Medical Entomology Laboratory, Entomology and Nematology Department, Institute of Food and Agricultural Sciences, University of Florida, Vero Beach, FL, USA; Florida Medical Entomology Laboratory, Entomology and Nematology Department, Institute of Food and Agricultural Sciences, University of Florida, Vero Beach, FL, USA; Florida Medical Entomology Laboratory, Entomology and Nematology Department, Institute of Food and Agricultural Sciences, University of Florida, Vero Beach, FL, USA; Florida Medical Entomology Laboratory, Entomology and Nematology Department, Institute of Food and Agricultural Sciences, University of Florida, Vero Beach, FL, USA; Puerto Rico Vector Control Unit, San Juan, PR, USA; Puerto Rico Vector Control Unit, San Juan, PR, USA; Florida Medical Entomology Laboratory, Entomology and Nematology Department, Institute of Food and Agricultural Sciences, University of Florida, Vero Beach, FL, USA

**Keywords:** invasive mosquito, Caribbean, island, dengue, species occurrence, IPM, IVM

## Abstract

As of October 2025, Puerto Rico has been experiencing an ongoing dengue outbreak that started in March 2024. The latest island-wide mosquito survey conducted in Puerto Rico during 2018 to 2019 covered 41 of the 78 municipalities and detected the presence of *Aedes aegypti* (Linnaeus, 1762) in 27 of the municipalities. Given the prolonged elevated circulation of the dengue virus on the island, we carried out an *Ae. aegypti* survey in June 2025 across 48 out of 78 municipalities. Here, we report the occurrence of *Ae. aegypti* in 43 out of 48 municipalities surveyed, some of which have not been reported in previous studies. Notably, 77.6% of cemeteries surveyed across 24 municipalities served as oviposition sites for *Ae. aegypti* and were found in 10 municipalities not previously recorded in literature. Due to observed frequency of shared larval habitat with *Ae. mediovittatus* (Coquillett, 1906), an analysis of co-occurrence patterns is provided from collected sites. In addition, updated distribution of *Ae. mediovittatus* is provided. The larvivorous *Tx. portoricensis* (Röder, 1885) was observed opportunistically, and co-occurrence analysis was performed to assess its potential for biocontrol. We also documented effective integrated pest management practices that minimize mosquito breeding where mosquito larvae were absent. Overall, the updated distribution of the three species in Puerto Rico reveals a broader distribution than previously reported, and co-occurrence analysis confirms that *Ae. aegypti* and *Ae. mediovittatus* frequently share larval habitat. This highlights the implications for dengue transmission risk and the need for continuous surveillance to support mosquito control efforts.

## Introduction

Accurate occurrence records of mosquito species are important for mosquito management and pathogen surveillance. Regular surveillance of mosquito species can enable monitoring of their distribution and abundance, allowing public health agencies to respond promptly to potential risks (Bakhiyi et al. 2024). Such data is also critical for evaluating the effectiveness of control programs and for developing future management strategies. Moreover, because environmental factors such as urbanization and climate change can increase vector abundance and risks of infection (Ryan et al. 2019, Wilke et al. 2021), regular surveillance is a key component of mosquito management strategies.

**Fig. 1.**
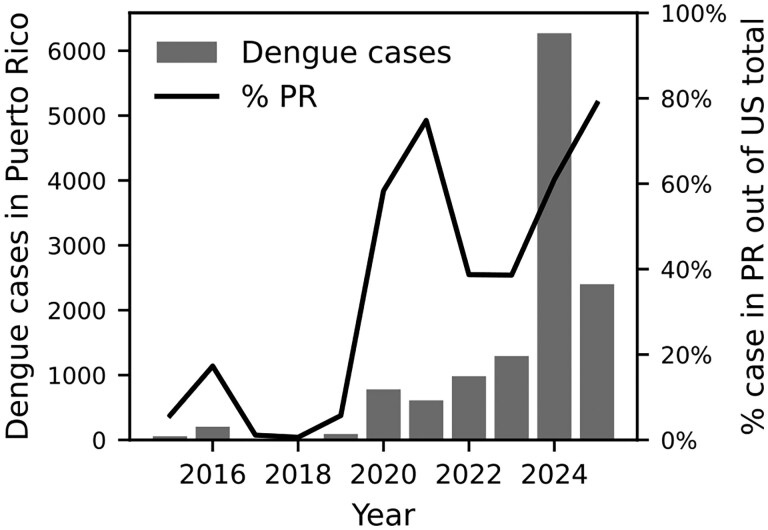
Dengue cases in Puerto Rico between 2015-2025 (gray bar) and % cases corresponding to Puerto Rico compared to the national case number (black line). Data is from CDC (CDC 2025).

As of October 2025, Puerto Rico has been experiencing an ongoing dengue outbreak that was declared in March 2024 (Ware-Gilmore et al. 2025). This region is currently experiencing the highest dengue case load in the United States (Fig. 1). Documented outbreaks in Puerto Rico have involved the co-circulation of multiple dengue serotypes (1 to 4) which exacerbates the health risks to residents (Rigau-Pérez et al. 2002, Sharp et al. 2019, Rodriguez et al. 2024). High circulation of dengue virus (DENV) in Puerto Rico can also spill over to other neighboring regions including the continental United States leading to high travel-related dengue cases as well as increasing the risk of local dengue transmission in neighboring states like Florida (Taylor-Salmon et al. 2024). Therefore, studying DENV and its vector species in Puerto Rico is not only beneficial for the residents in Puerto Rico but also for population in the neighboring areas and the whole nation.

The invasive species, *Aedes aegypti*, serve as main vector for DENV in Puerto Rico. *Aedes albopictus*, another invasive species and vector of DENV, was thought to be introduced to Puerto Rico before 2005 (Cook et al. 2006); however, it was not observed in the multiple island-wide mosquito species surveys in following years (Barrera et al. 2011, 2012, Yee et al. 2021). *Aedes mediovittatus* is a native species that has shown high competency for transmission (Gubler et al. 1985, Tomashek et al. 2009, Poole-Smith et al. 2015). Previous studies have reported that, *Ae. mediovittatus* is an efficient dengue vector, demonstrating a capacity for transmission comparable to *Ae. aegypti* in laboratory conditions (Gubler et al. 1985, Poole-Smith et al. 2015). In addition, *Ae. mediovittatus* has been suggested to be a maintenance vector that sustains dengue transmission between outbreaks, likely due to its high efficiency in vertical transmission (Gubler et al. 1985). Both species have also been suggested to co-occur in low-density urban, suburban and rural areas (Cox et al. 2007, Little et al. 2011), demonstrating the importance of understanding current distribution and co-occurrence patterns to gain further insight on dengue transmission risks and informing vector surveillance efforts.

A recent island-wide survey encompassed 41 municipalities in Puerto Rico documenting the presence of *Ae. aegypti* from 27 municipalities and *Ae. mediovittatus* in 19 municipalities (Yee et al. 2021). Fourteen of the 41 municipalities (34%) harbored both *Ae. aegypti* and *Ae. mediovittatus*. The survey also observed *Ae. aegypti* co-occurring with *Tx. portoricensis* in Utuado, Puerto Rico, (Yee et al. 2021). Another recent study investigated the presence of mosquito larvae in over 9000 water-holding containers from 16 cemeteries across six municipalities (Caguas, Humacao, Juncos, Las Piedras, Naguabo, and Yabucoa) in the east region of Puerto Rico between 2019 and 2020 (Otero et al. 2022). *Aedes aegypti* and *Ae. mediovittatus* were identified as the most abundant species. The two species accounted for 84.9% of all collected immature mosquitoes in the six municipalities surveyed. *Aedes aegypti* was present in every cemetery except for Municipal Ramon Delgado (Juncos) and Vale de Paz (Las Piedras). *Aedes mediovittatus* was identified in every cemetery except Borinquen Memorial I (Caguas), Valle de Paz (Las Piedras) and La Inmaculada (Juncos).

During our investigation, checking prior species occurrence records of *Ae. aegypti* in Puerto Rico using Global Biodiversity Information Facility (GBIF) and other publications, we discovered that 20 municipalities out of 78 municipalities in Puerto Rico had no records of the presence of *Ae. aegypti* (Yee et al. 2021, Otero et al. 2022, GBIF Secretariat 2023). Given the ongoing dengue outbreak in Puerto Rico, we carried out an additional survey to better inform municipalities for vector control efforts. Concurrently, due to potential vector competence for dengue and shared larval habitat of the native of *Ae. mediovittatus*, a secondary objective was to investigate its updated geographic records and co-occurrence pattern with *Ae. aegypti*. We also report on the opportunistic detection of *Tx. portoricensis* found in shared larval habitats with *Ae. aegypti*, which may offer insights as a potential biocontrol agent.

## Materials and Methods

### Mosquito Collection

We collected both adult and immature stages (larvae and pupae) of mosquitoes from 48 municipalities (62%) in Puerto Rico covering 113 locations in June of 2025 during the wet season (Supplementary Table S1). We set up BG-Sentinel (BG-S) traps (Biogents AG, Regenburg, Germany) in residential areas with permission from homeowners. An average of 1 to 2 BG-S traps were set per household. Traps were set to run for 24 to 36 h. Mosquitoes in BG-S collection bags were brought to a laboratory and kept in −20 °C until species identification by morphological examination.

**Fig. 2.**
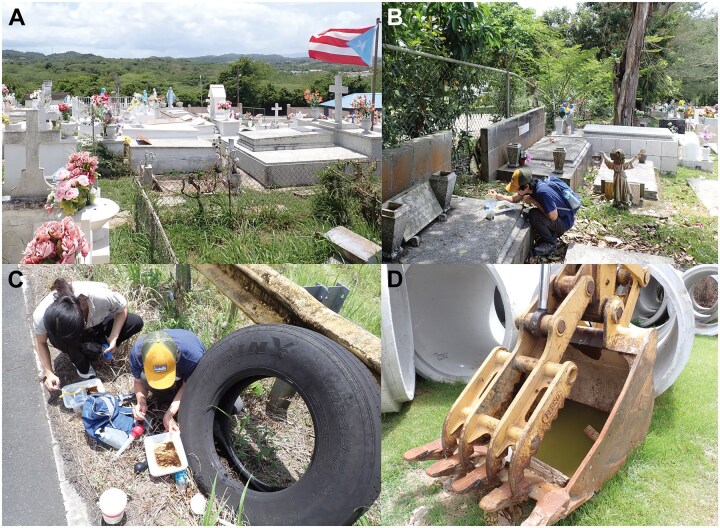
Example habitat photos. A) a cemetery with flower vases. B) a cemetery with flower vases and more shade. C) discarded tire holding water. D) excavator bucket holding water.

Flower vases in cemeteries (Fig. 2A and B), discarded tires on the roadside (Fig. 2C), and other containers holding water (Fig. 2D) were examined for the presence of immature stages (larvae and pupae). We examined multiple locations within a cemetery for mosquito larvae and samples were collected from one to six locations within each cemetery. The samples were extracted using turkey basters, a ladle, and/or plastic transfer pipettes. The larvae and pupae were held in Whirl-Pak plastic bags (Whirl-Pak, Austin, Texas, United States) for transport between collection sites and the base of operation, which was either Puerto Rico Vector Control Unit (PRVCU) laboratory in San Juan or Ponce. GPS coordinates of each trap and larval site were recorded using Google Maps app on mobile devices. The coordinates of residential address have been adjusted to the nearest 10 s for privacy purpose. For larvae sampled from multiple vases within two-meter radius were stored in one bag with one GPS coordinate. Larvae were put into containers separated by coordinates and left until pupation. Pupae were transferred into 7-dram vials with water for emergence. The eclosed adults were frozen in −20 °C freezer before species identification. All samples were preserved in 70% ethanol for DNA extraction.

### Species Identification

Species identification of adult mosquitoes was determined by morphology using the in-house identification keys provided by the PRVCU. Concurrently, IDX device (Vectech, Baltimore, Maryland, United States), machine learning-based *Ae. aegypti* identification version 6.0.0, was used to separate *Ae. aegypti* from non-target species more efficiently. This device has been used in other studies to identify *Ae. aegypti* and showed 88.8% accuracy at version 4.0 (Gupta et al. 2024). We accepted *Ae. aegypti* species calls if the confidence level is above 80%. The samples with less confident (<80%) or unknown calls were further examined for morphological features to confirm its species according to PRVCU identification keys. Samples we could not confidently confirm were stored in 70% alcohol for molecular species identification.

Larvae, pupae, and damaged adult specimens were identified to species using multiple molecular assays. First, samples were assessed for *Ae. aegypti* by an internal transcribed spacer 2 (ITS2) PCR assay developed by Menegon et al. (2025) with modified set of primers using Aedes-F2 (5ʹ-AGG ACA CAT GAA CAC CGA CA-3ʹ), JAP-R (5ʹ-TAT ACT ACG CTG CCG AGA GG-3ʹ), and AEG-R2 (5ʹ-TGA GTG AAT GAT GGA ATA CAA CA-5ʹ) primers. *Aedes albopictus* primers of Menegon et al. (2025) were not included because they repeatedly failed to amplify for positive control. A 25 µL of PCR mixture was prepared for each sample to contain 1 µL of extracted DNA template, 12.5 µL of 2X OneTaq Master mix with buffer (Life Technologies, Carlsbad, CA), 0.05 µL each primer in 10 µM (final concentration 10 pmol per primer), and 11.3 µL of PCR-grade water to amplify ITS2 region. DNA was initially denatured at 94 °C for 5 min, followed by 40 cycles of denaturation at 94 °C for 30 s, annealing at 50.2 °C for 30 s, and extension at 72 °C for 60 s. Then a final extension step of 72 °C was set for 5 min before the PCR products were held at 4 °C before storage at −20 °C. The PCR products were stained with SYBR^TM^ Safe DNA Gel Stain (Invitrogen, Waltham, Massachusetts, United States) and separated by its fragment size by electrophoresis on a 1.5% agarose gel.

For samples with no amplification on *Aedes* ITS2 PCR assay (Menegon et al. 2025), we amplified cytochrome c oxidase I (COI) sequence using DNA Barcoding primers LCO1490 (5ʹ-GGT CAA CAA ATC ATA AAG ATA TTG G-3ʹ) and HCO2198 (5ʹ-TAA ACT TCA GGG TGA CCA AAA AAT CA-3ʹ) (Hebert et al. 2003). PCR protocol followed the method described in Reeves et al. (2021). The PCR product was sent to Eurofins for Sanger Sequencing. The resulting DNA sequences were entered into Basic Local Alignment Search Tool—nucleotide (BLASTn) to find a matching species (Altschul et al. 1990).

### Co-Occurrence Analysis

Locations were standardized by consolidating each surveyed point ∼1 km apart within each municipality into a single location with either presence or absence of *Ae. aegypti, Ae. mediovittauts* and *Tx. portoricensis*. A co-occurrence analysis was conducted to evaluate the co-occurrence of the three mosquito species using *cooccur* package ver. 1.3 (Griffith et al. 2016). The co-occurrence analysis assumed independent species occurrences as a null hypothesis and evaluated each species pair by comparing observed and expected co-occurrence frequencies. This analysis utilizes qualitative presence/absence data (Griffith et al. 2016). All statistical analyses were performed using R version 4.5.0 (Griffith et al. 2016, R Core Team).

### Data Analysis

Maps of sampling locations and species occurrence records were generated using QGIS version 3.40.4 (QGIS 2025). The shapefile of Puerto Rico municipalities was obtained from Datos.PR (Datos.PR 2018). For data reporting purposes, we used six regions–namely Metro, East, North, Central, South, and West regions—commonly referred to in governing and tourism (Rivera 2025) for aggregating municipality data. We used GBIF (GBIF Secretariat 2023), Yee et al. (2021), and Otero et al. (2022) records to determine municipality records that were new in Puerto Rico. We also used the six United States Geological Survey climatic subdivisions in Puerto Rico (USGS CFWSC 2016) to examine any trends in association with climatic conditions and mosquito species distribution.

## Results

### *Aedes aegypti* Occurrence in Puerto Rico

**Fig. 3.**
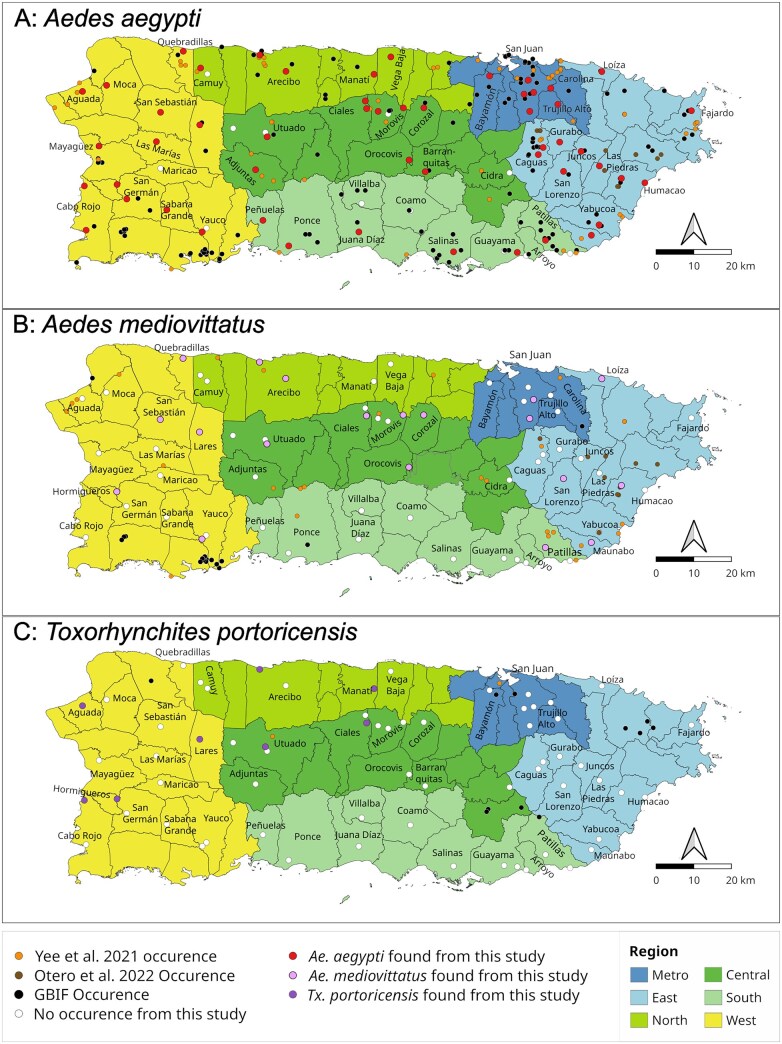
Species occurrence records. A) *Ae. aegypti* (red dots). B) *Ae. mediovittatus* (pink dots). C) *Tx. portoricensis* (purple dots) from this study. Black dots indicate GBIF occurrence record as of June 2025, orange dots indicate occurrence records from Yee *et al*. (2021), brown dots indicate occurrence records from Otero *et al*. (2022). Metro region is marked in blue, East region in light blue, North region in green, Central region in dark green, South region in lime green, and West region in yellow background color.

The survey conducted in June 2025 across 48 municipalities over two weeks revealed that adult or immature stages of *Ae. aegypti* were present in 42 municipalities (Fig. 3A). Our survey includes 10 municipality records of *Ae. aegypti* occurrence (marked with asterisk (*) in Table 1) that were not previously recorded in literature. We did not collect any mosquitoes from Maricao in the Western region, Cidra in the Central region, and, Coamo and Villalba in the Southern region.

**Table 1.** Collection records of *Ae. aegypti, Ae. mediovittatus*, and *Tx. portoricensis* in municipalities of Puerto Rico during June 2025. The adult and larvae/pupae columns indicate the life stage at time of collection in the field. Asterisk (*) indicates new municipality record of *Ae. aegypti* occurrence. The summary table is provided here for brevity and detailed sample metadata are provided in supplemental Table S1

	*Ae. aegypti*			*Ae. mediovittatus*			*Tx. portoricensis*		
Municipalities	Adult	Larvae	Pupae	Adult	Larvae	Pupae	Adult	Larvae	Pupae
**Metro**	**42**	**25**	**7**	**4**	**6**	**5**			
**Bayamón**	5			0					
**San Juan**	28	25	7	4	6	5			
**Trujillio Alto**	9								
**East**	**72**	**65**	**5**	**3**	**3**				
**Caguas**	5								
**Fajardo**	30								
Gurabo*	1	5							
**Humacao**	14			1					
**Juncos**		34	1						
**Las Piedras**	1	4							
**Loíza**	17			1					
Maunabo*		6	4		3				
**San Lorenzo**	4			1					
**Yabucoa**		16							
**North**	**31**	**72**	**8**	**1**	**1**	**1**	**1**		**2**
**Arecibo**	6	40	5	1	1	1	1		1
**Camuy**	6								
**Manati**	5	32	3						2
Vega Baja*	14								
**Central**	**15**	**54**	**14**	**1**	**13**	**12**		**2**	**1**
**Adjuntas**	2								
**Barranquitas**	5								
**Ciales**	1	29	3		5	7		1	1
**Corozal**		1	7			5			
**Morovis**		1	3		1				
Orocovis*	6			1					
**Utuado**	1	23	1		7			1	
**South**	**29**	**21**	**10**			**1**			
**Guayama**	15								
**Juana Díaz**	2								
**Patillas**		15	9			1			
Peñuelas*		6	1						
**Ponce**	8								
**Salinas**	4								
**West**	**30**	**175**	**9**	**2**	**18**	**25**		**2**	**2**
**Aguada**	1	7	1					1	
**Cabo Rojo**	6	27						1	
Hormigueros*		1			2	1			1
**Lares**	3	13	1		5	23			1
Las Marías*		51	6						
**Mayagüez**	3								
Moca*		15	1						
**Quebradilla**	11			1					
Sabana Grande*		12							
**San Germán**		38							
San Sebastián*	1	11			11	1			
**Yauco**	5			1					
**Total**	**219**	**412**	**53**	**11**	**41**	**44**	**1**	**4**	**6**

The majority of larval sites we examined were in cemeteries (41/54 = 75.9%). Collections from water held in tires (*n* = 7 in 5 municipalities) were serendipitous encounters as we traveled between municipalities and thus occupy relatively small portions of our larval collections. *Aedes aegypti* were found in 77.6% of the larval collection sites.

### *Aedes albopictus* Occurrence in Puerto Rico

We did not collect any *Ae. albopictus* during our mosquito survey.

### *Aedes Mediovittatus* Occurrence in Puerto Rico

*Aedes mediovittatus* was found in 26 locations in 17 municipalities (Fig. 3B). Twelve of the 17 municipalities (Ciales, Corozal, Hormigueros, Humacao, Lares, Loíza, Orocovis, Quebradillas, San Lorenzo, San Sebatian, Utuado, and Yauco) are new municipality records (Yee et al. 2021, Otero et al. 2022, GBIF Secretariat 2023). This species was most commonly found in the West region (8/26 = 30.8%) and least common in South region (1/16 = 6.3%; Table 2). With respect to USGS Climatic subdivision (USGS CFWSC 2016), *Ae. mediovittatus* was most common in the North Coastal (6/20 = 30%) and Western Interior (11/36 = 30.6%) region. Catch numbers using BG-S traps were generally low (*n* = 0 to 4 per trap). *Aedes mediovittatus* are often found together with *Ae. aegypti* (22/25 = 88%). In comparison, cooccurrence of *Ae. mediovittatus* and *Ae. aegypti* were previously noted at 77% (10/13, Otero et al. 2022) and 1.76% (13/74, Yee et al. 2021).

**Table 2.** The number of locations where *Ae. mediovittatus* was detected grouped by Region or USGS Climatic subdivision (2016)

Region	Total collection locations	*Ae. mediovittatus* positive locations	% positive	USGS Climatic subdivision (2016)	Total collection locations	*Ae. mediovittatus* positive locations	% positive
**Metro**	16	4	25.0%	Eastern Interior	11	1	9.1%
**Central**	24	6	25.0%	North Coastal	20	6	30.0%
**East**	21	5	23.8%	Northern Slopes	14	2	14.3%
**North**	10	2	20.0%	South Coastal	6	1	16.7%
**South**	16	1	6.3%	Southern Slopes	26	5	19.2%
**West**	26	8	30.8%	Western Interior	36	11	30.6%

### *Toxorhynchites Portoricensis* Occurrence in Puerto Rico

*Toxorhynchites portoricensis* was found in nine locations across eight municipalities from our study (Fig. 3C). These include Ciales and Utuado in the Central region; Arecibo and Manatí in the North region; and Aguada, Hormigueros, Cabo Rojo, and Lares in the West region. Seven of the eight municipalities (Aguada, Arecibo, Cabo Rojo, Ciales, Lares, Hormigueros, and Manatí) are new municipality records for this species. Previously, occurrence of this species was noted in Bayamón, Cayey, Isabela, Luquillo, Patillas, Río Grande, San Juan, and Utuado municipalities (Yee et al. 2021, GBIF Secretariat 2023). Collectively, this species has been found in all regions except the South region (Fig. 3C, Table 3). With respect to USGS Climatic subdivision (USGS CFWSC 2016), this species was most common in Northern Slopes (14.3%) followed by Western Interior (11.1%) and Southern Slopes (7.7%). We did not encounter this species in the Eastern Interior or South Coastal region.

**Table 3.** The number of locations where *Tx. portoricensis* was detected grouped by Region or USGS Climatic subdivision (2016)

Region	Total collection locations	*Tx. portoricensis* positive locations	% positive	USGS climatic subdivision (2016)	Total collection locations	*Tx. Portoricensis* positive locations	% positive
**Metro**	16	0	0.0%	Eastern interior	11	0	0.0%
**Central**	24	3	12.5%	North coastal	20	1	5.0%
**East**	21	0	0.0%	Northern slopes	14	2	14.3%
**North**	10	2	20.0%	South coastal	6	0	0.0%
**South**	16	0	0.0%	Southern slopes	26	2	7.7%
**West**	26	4	15.4%	Western interior	36	4	11.1%

### Co-Occurrence Analysis

After consolidating the occurrence data from nearby (<1 km radius) locations, a total of 65 occurrence data for three mosquito species remained and were used in co-occurrence analysis (Supplementary Table S2). The co-occurrence analysis identified one significant positive association among the three species examined. *Aedes aegypti* and *Ae. mediovittatus* pair showed a significantly higher than expected frequency of co-occurrence (observed = 19, expected = 15.5, *P* = 0.0097). In contrast, non-significant co-occurrence frequencies were shown in *Ae. aegypti* and *Tx. portoricensis* pair (observed = 7, expected = 6.5, *P* > 0.54) and *Ae. mediovittatus* and *Tx. portoricensis* pair (observed = 4, expected = 2.3, *P* > 0.17). Overall, only the *Ae. aegypti* and *Ae. mediovittatus* exhibited a positive association, while all other species pairs had random co-occurrence patterns.

### Successful Cases of Integrated Vector Management in Puerto Rico

**Fig. 4.**
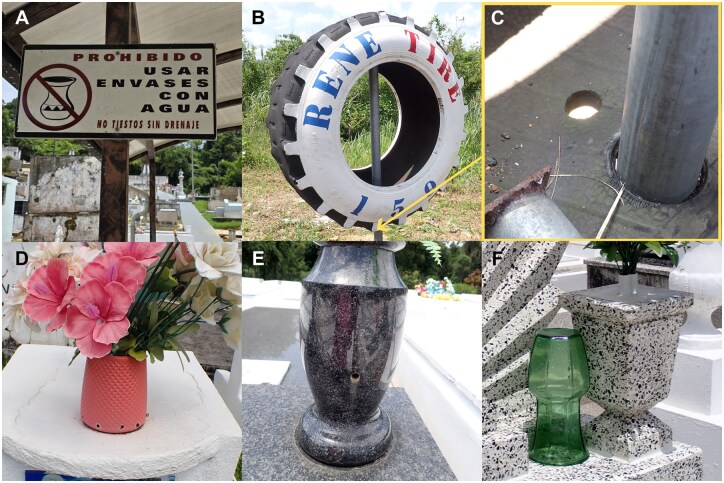
Examples of management practices from locations without any mosquitoes. A) Sign displayed at the entrance of the cemetery in Maricao prohibiting the use of containers without drainage that can hold water. B) Tire landmark C) with hole drilled on the base of the inside of the tire for drainage. D) Flower vase with holes on the base for drainage. E) Stone flower vase with drainage provided by the cemetery. F) Use of faux flowers with pot cemented and vase left upside down to prevent accumulation of stagnant water.

There were three locations where mosquito presence was not detected during our collection visiting 116 different locations (Supplementary Table S1). Two were cemeteries in Maricao and Morovis municipalities and one tire shop in Morovis. The entrance of the Maricao cemetery had multiple clear signs banning the use of flower vases in the cemetery (Fig. 4A). Communication with the Maricao cemetery manager revealed that the prevention efforts were a result of prior collaboration with PRVCU, during which potential breeding habitats and management practices were discussed. The impact of the collaboration was evident as active management was noted in minimizing creation of pools of water in the cemetery. These included vases filled to the brim with sand or dirt, vases with drainage holes, and the practice of turning vases upside down when not in use (Fig. 4D to F); this practice was also noted in the cemetery in Morovis. Prevention at the tire shop included preventing buildup of water by shipping out used tires weekly and adding holes into a shop sign made from a used tire (Fig. 4B and C).

## Discussion

### Distribution of Dengue Vector in Puerto Rico

During the ongoing dengue outbreak in Puerto Rico, we were able to observe *Ae. aegypti* from 43 municipalities out of 44 total municipalities surveyed in June 2025. Our records included 10 municipality records that did not previously report the presence of *Ae. aegypti*. While it is widely held belief that this species is widespread in Puerto Rico, this is not a given fact and varies depending on the collection method, timing of collection, location, and protocol as evident by Yee et al. (2021) which observed *Ae. aegypti* from 27 municipalities out of 48 total surveyed. The ubiquitous occurrence of *Ae. aegypti* indicates that this species does not have limiting environmental conditions within Puerto Rico. The collecting period of this study was the early stage of the rainy season, and we anticipate that more locations will become available for *Ae. aegypti* reproduction in the later period of the year around late September and early October based on the past trends in dengue cases (Salud 2025).

From our survey of 113 locations in 48 municipalities, we did not find *Ae. albopictus*. The presence of *Ae. albopictus* in Puerto Rico was first reported in 2006 (Cook et al. 2006). Since this initial report, the subsequent extensive surveys have not found *Ae. albopictus* on the main island of Puerto Rico (Barrera et al. 2011, 2012, Yee et al. 2021). In the southernmost municipalities of the West region, GBIF recorded with concrete dates of observation reports *Ae. albopictus* between 2016 and 2020 from Lajas and Guánica municipalities (GBIF Secretariat 2023). However, as these two municipalities were not included in this study, we were unable to verify the records. Therefore, the presence of *Ae. albopictus* in Puerto Rico needs further investigations to verify.

### Other Mosquitoes Sharing Oviposition Sites with *Ae. aegypti*

*Aedes mediovittatus* is a native mosquito species in Puerto Rico and shares oviposition sites with *Ae. aegypti* (Little et al. 2011, Otero et al. 2022)*. Aedes aegypti* has been frequently reported to outcompete other mosquito species in interspecific interactions (Santana-Martínez et al. 2017, Lushasi et al. 2024). Nevertheless, *Ae. mediovittatus* still coexists with *Ae. aegypti* in shared habitats, possibly because *Ae. mediovittatus* exhibits a competitive advantage over *Ae. aegypti* (Yee et al. 2025). Another possible explanation is that environmental urban gradient (ie vegetation vs impervious coverage) and high resource availability influence competition between the two species. A study conducted in San Juan, Puerto Rico, across a gradient of environmental conditions reported a positive association between high resource availability and the coexistence of *Ae. aegypti* and *Ae. mediovittatus* in low impervious regions with high canopy (Reyes-Torres et al. 2025). While it was not possible to evaluate the competitive relationships between *Ae. aegypti* and two other species in this study, because quantitative collections were not conducted, we were still able to observe a significant pattern of coexistence between *Ae. aegypti* and *Ae. mediovittatus* through co-occurrence analysis. These co-occurrences were observed mostly in vases in cemeteries with scattered trees and away from residential areas consistent with past studies in which overlap were suggested to be common in low density urban to rural settings with vegetation (Cox et al. 2007, Little et al. 2011, Reyes-Torres et al. 2025).

*Aedes mediovittatus* are continuously monitored for DENV infection by PRVCU because they are often collected with *Ae. aegypti.* Literature has shown *Ae. mediovittatus* to be competent vectors of DENV-1, DENV-2, and DENV-3, on par with *Ae. aegypti*, but less susceptible to DENV-4 (Poole-Smith et al. 2015). In laboratory, *Ae. mediovittatus* was able to transmit DENV to mice through vertical transmission, suggesting that *Ae. mediovittatus* are efficient vectors and may play a significant role in the circulation of DENV between outbreaks (Gubler et al. 1985).

While *Ae. aegypti* has many studies investigating its immune responses against DENV infection with good genomic resources (Behura et al. 2011, Castillo-Méndez and Valverde-Garduño 2020), little is known about *Ae. mediovittatus.* There are very limited genetic resources for *Ae. mediovittatus* with only two COI sequences available as of September 2025 (Gibson et al. 2012). While it will take a long time to build equivalent genetic resources for *Ae. mediovittatus* for comparative genomics/transcriptomics studies, the comparison of immune responses against DENV infection between *Ae. mediovittatus* and *Ae. aegypti* could illuminate mechanisms of virus replication in these mosquito species. The understanding of virus transmission in two different mosquitoes with varying degrees of vector competency can be utilized to block virus transmission in mosquitoes in the future.

*Toxorhynchites portoricensis*, belonging to the subgenus *Lynchiella*, is the only *Toxorhynchites* species known to occur in Puerto Rico. This study identified additional distribution of the species than previously recorded, documenting occurrence in seven new municipalities. Past studies observed occurrence in moist forest, lower montane wet forest and subtropical wet forests at both low and high elevation (Torres-Valcárcel et al. 2014, Yee et al. 2021, GBIF Secretariat 2023). Our survey likewise recorded the species in similar climatic environments, including moist climate (Aguada, Arecibo, Cabo Rojo, Cilaes, Hormigueros, and Manatí) and subtropical wet forest (Lares and Utuado) (Fig. 3C). While some species (such as *Tx. splendens* and *Tx. amboinensis*) prefer coastal habitats, *Toxorhynchites* typically prefer sylvatic habitats near forests (Steffan and Evenhuis 1985, Focks 2007, Donald et al. 2020, Ceretti-Junior et al. 2024). We observed *Tx. portoricensis* occurring in both coastal and forested regions.

*Toxorhynchites* genus is typically found in locations like discarded tires and tree holes (Schreiber 2007). In Brazil, *Tx. portoricensis* was exclusively found in artificial larval sites such as plastic cups and bottles (Ceretti-Junior et al. 2024). We found *Tx. portoricensis* in artificial containers like tires, flower vases from a cemetery in Ciales and in water collected in an excavator scoop in the residential area in Cabo Rojo (Fig. 2D). Due to our primary goal of collecting *Ae. aegypti*, we only looked at artificial containers and collection of *Tx. portoricensis* was our bycatch. Therefore, while our study agrees with Ceretti-Junior et al. 2024, we cannot rule out the possibility of *Tx. portoricensis* in Puerto Rico utilizing natural containers for oviposition.

During our field work, we observed *Tx. portoricensis* larvae consuming their own species in the same water. The carnivorous larva of this genus consumes other mosquito larvae and has long been considered for biological control options as part of the integrated vector management (Focks 2007, Albeny et al. 2011, Seok et al. 2022). *Aedes aegypti* in particular are attracted to the bacterial makeup of waters already predated by *Toxorhynchites theobaldi*, making *Toxorhynchites* a more beneficial contender as biocontrol agent (Albeny-Simões et al. 2014). One of the characteristic behaviors in some *Toxorhynchites* species is the prepupal killing of surrounding larvae without consuming them before becoming pupae (Focks 2007). While this behavior has not been observed in this species yet, it might represent another possible way by which it suppresses other mosquitoes. Although *Tx. portoricensis* may have the potential to reduce population sizes, its presence did not show a significant negative association with *Ae. aegypti* and *Ae. mediovittatus* in the co-occurrence analysis. This lack of significance may be due to lack of occurrence data from this study. Alternatively, this may also be explained by predator-mediated oviposition avoidance, as female mosquitoes are capable of detecting predator cues and selecting safer habitats for larvae (Wasserberg et al. 2013). Moreover, differences in habitat size preference between the two species may also influence low encounter rate (Sunahara et al. 2002). Additional surveillance is needed to better document occurrence and run a comprehensive co-occurrence analysis to determine feasibility of this species as a biocontrol agent.

### Integrated Vector Management to Reduce *Ae. aegypti*

Every municipality has at least one cemetery in Puerto Rico, often located in one of the major towns in the municipality adjoining the large residential area. It is a permanent fixture in their landscape and often has dedicated maintenance staff managing the ground. Management practices, as seen at the cemetery in Morovis and Maricao, show that appropriate proactive management could contribute to mosquito control. The management practices observed as shown in Fig. 4 could be effective in discouraging larval habitats. Focused education on the maintenance and property management personnel on the permanent infrastructure like cemeteries could have lasting impact in reducing the mosquito reproduction in Puerto Rico. This practice can also apply in other continental US where cemeteries serve as mosquito breeding source (Vezzani 2007, Champion and Vitek 2014, Wilke *et al*. 2020).

## Supplementary Material

tjag048_Supplementary_Data
