# Supplementary material for: New distribution records of Aedes aegypti (Linnaeus), Aedes mediovittatus (Coquillett), and Toxorhynchites portoricensis (Röder) (Diptera: Culicidae) in Puerto Rico and their relevance to integrated vector management
Source: J Med Entomol. 2026 Mar 27;63(2):tjag048. doi: 10.1093/jme/tjag048 (PMC13023038; doi:10.1093/jme/tjag048)
Supplement: tjag048_Supplementary_Data [file tjag048_supplementary_data.zip › 23-Mar-2026_112118_Supplementary_Material.zip!/Table S2. Consolidated records data for co-occurence analysis.docx]

| **Region** | **Municipality** | **Location ID** | **Location type** | **Collection method** | ***Ae. aegypti*** | ***Ae. mediovittatus*** | ***Tx. portoricensis*** |
| --- | --- | --- | --- | --- | --- | --- | --- |
| Central | Adjuntas | CAD | residential | BG-Sentinel | O | X | X |
| Central | Barranquitas | CBA | residential | BG-Sentinel | O | X | X |
| Central | Ciales | CCI1 | cemetery | Larval collection | O | O | O |
| Central | Ciales | CCI2 | cemetery | Mouth Aspirator | O | X | X |
| Central | Cidra | CCID | residential | BG-Sentinel | X | X | X |
| Central | Corozal | CCOR | cemetery | Larval collection | O | O | X |
| Central | Morovis | CMO1 | tire | Larval collection | O | X | X |
| Central | Morovis | CMO2 | cemetery | Larval collection | X | X | X |
| Central | Morovis | CMO3 | tire | Larval collection | O | O | X |
| Central | Orocovis | COR | residential | BG-Sentinel | O | O | X |
| Central | Utuado | CUT1 | cemetery | Larval collection | O | O | X |
| Central | Utuado | CUT2 | tire | Larval collection | X | X | O |
| Central | Utuado | CUT3 | cemetery | Larval collection | X | X | X |
| East | Caguas | ECA1 | residential | BG-Sentinel | O | X | X |
| East | Caguas | ECA2 | residential | BG-Sentinel | O | X | X |
| East | Fajardo | EFA | residential | BG-Sentinel | O | X | X |
| East | Gurabo | EGU | cemetery | Larval collection | O | X | X |
| East | Humacao | EHU1 | residential | Larval collection | O | X | X |
| East | Humacao | EHU2 | residential | Larval collection | O | O | X |
| East | Juncos | EJU | cemetery | Larval collection | O | X | X |
| East | Las Piedras | ELP | residential, cemetery | BG-Sentinel, Larval collection | O | X | X |
| East | Loiza | ELO | residential | BG-Sentinel | O | O | X |
| East | Maunabo | EMA | cemetery | Larval collection | O | O | X |
| East | San Lorenzo | ESL | residential | BG-Sentinel | O | O | X |
| East | Yabucoa | EYA | cemetery | Larval collection | O | X | X |
| Metro | Bayamon | MBA | residential | BG-Sentinel | O | X | X |
| Metro | San Juan | BGCA | residential | BG-Sentinel | O | X | X |
| Metro | San Juan | MSJ1 | residential | BG-Sentinel | O | O | X |
| Metro | San Juan | MSJ2 | residential | BG-Sentinel | O | X | X |
| Metro | San Juan | MSJ3 | cemetery | Larval collection | O | O | X |
| Metro | San Juan | MSJ4 | residential | BG-Sentinel | O | X | X |
| Metro | San Juan | MSJ5 | residential | Mouth Aspirator | O | X | X |
| Metro | Trujillio Alto | MTA | residential | BG-Sentinel | O | X | X |
| North | Arecibo | NAR1 | residential | Larval collection | O | O | X |
| North | Arecibo | NAR2 | residential | BG-Sentinel | O | O | O |
| North | Camuy | NCA1 | forest | BG-Sentinel | X | X | X |
| North | Camuy | NCA2 | residential | BG-Sentinel | O | X | X |
| North | Manati | NMA | residential | BG-Sentinel, Larval collection | O | X | O |
| North | Vega Baja | NVB | residential | BG-Sentinel | O | X | X |
| South | Arroyo | SAR | cemetery | Larval collection | X | X | X |
| South | Coamo | SCO | city | BG-Sentinel | X | X | X |
| South | Guayama | SG1 | residential | BG-Sentinel | O | X | X |
| South | Guayama | SG2 | cemetery | Larval collection | X | X | X |
| South | Juana Diaz | SJD | residential | BG-Sentinel | O | X | X |
| South | Patillas | SPA1 | cemetery | Larval collection | O | O | X |
| South | Patillas | SPA2 | cemetery | Larval collection | X | X | X |
| South | Penuelas | SPE | cemetery | Larval collection | O | X | X |
| South | Ponce | SPO | residential | BG-Sentinel | O | X | X |
| South | Salinas | SSA | residential | BG-Sentinel | O | X | X |
| South | Villalba | SVI | residential | BG-Sentinel | X | X | X |
| West | Aguada | WAG | cemetery | Larval collection | O | X | O |
| West | Cabo Rojo | WCB1 | cemetery | Larval collection | O | X | X |
| West | Cabo Rojo | WCB2 | residential | Larval collection | O | X | O |
| West | Hormigueros | WHO | tire | Larval collection | O | O | O |
| West | Lares | WLA | cemetery | Larval collection | O | O | O |
| West | Las Marias | WLM | cemetery | Larval collection | O | X | X |
| West | Maricao | WMA | cemetery | Larval collection | X | X | X |
| West | Mayaguez | WMAY | residential | BG-Sentinel | O | X | X |
| West | Moca | WMO | cemetery | Larval collection | O | X | X |
| West | Quebradilla | WQU | residential | BG-Sentinel | O | O | X |
| West | Sabana Grande | WSGA | cemetery | Larval collection | O | X | X |
| West | San German | WSGE | tire | Larval collection | O | X | X |
| West | San Sebastian | WSS | cemetery | Larval collection | O | O | X |
| West | Yauco | WAY1 | cemetery | Larval collection | X | X | X |
| West | Yauco | WAY2 | residential | BG-Sentinel | O | O | X |
